# Celastrol Protects against Cerebral Ischemia/Reperfusion Injury in Mice by Inhibiting Glycolysis through Targeting HIF-1*α*/PDK1 Axis

**DOI:** 10.1155/2022/7420507

**Published:** 2022-01-05

**Authors:** Mengyuan Chen, Maozhu Liu, Ying Luo, Jun Cao, Fanning Zeng, Lu Yang, Junqing Yang, Tao Tao, Yu Jiang

**Affiliations:** ^1^Department of Anaesthesiology, Central People's Hospital of Zhanjiang, 236 Yuanzhu Road, Zhanjiang, Guangdong 524045, China; ^2^College of Pharmacy, Chongqing Medical University, Chongqing Key Laboratory of Biochemistry and Molecular Pharmacology, Chongqing 400016, China; ^3^Department of Anaesthesiology, Nanfang Hospital, Southern Medical University, Guangzhou, Guangdong 510515, China

## Abstract

Cerebral ischemia/reperfusion (I/R) injury is closely related to dysfunctional glucose metabolism. Celastrol is a bioactive compound that has been found to exhibit neuroprotective effects in cerebral ischemia, while whether it can protect against cerebral I/R injury by regulating glycolysis remains unclear. The goal of this study is to investigate the role of celastrol on cerebral I/R injury and its underlying mechanisms in transient middle cerebral artery occlusion (tMCAO) mice. *Methods*. To observe the protective effect of celastrol and select its optimal dosage for further study, neurological score, TTC staining, and HE staining were used to evaluate neurological function, cerebral infarct volume, and cortical cell damage, respectively. QRT-PCR and Western blot were used to detect the mRNA and protein expression of hypoxia inducible factor-1*α* (HIF-1*α*), pyruvate dehydrogenasekinase1 (PDK1), lactate dehydrogenase A (LDHA), glucose transporter1 (GLUT1), and hexokinase2 (HK2), respectively. The lactate production, ATP level, and glucose content were assessed by assay kits. *Results*. Our results indicated that celastrol dose-dependently improved neurological function and reduced cerebral infarct volume and cortical cell death of tMCAO mice, and its optimal dosage was 4.5 mg/kg. In addition, celastrol significantly blocked I/R-induced increase of LDHA, GLUT1, HK2, and lactate production as well as decrease of ATP level and glucose content. Moreover, celastrol inhibited the I/R-induced upregulation of HIF-1*α* and PDK1. Overexpression of HIF-1*α* by DMOG reversed the protective effect of celastrol on cerebral I/R injury and blocked celastrol-induced suppression of glycolysis. *Conclusions*. Taken together, these results suggested that celastrol protected against cerebral I/R injury through inhibiting glycolysis via the HIF-1*α*/PDK1 axis.

## 1. Introduction

Stroke is one of the major causes of disability and death around the world and brings heavy burden to the society [1, 2]. According to estimates, approximately 87% of all strokes are ischemic strokes characterized by reduction in cerebral blood supply; the remaining 13% of strokes are hemorrhagic strokes [3]. Currently, the use of recombinant tissue plasminogen activator (r-tPA) to quickly restore the cerebral blood supply is an effective strategy for relieving ischemic brain injury [4, 5]. However, reperfusion of blood flow to the ischemic brain tissue can further aggravate brain damage after cerebral ischemia, which is regarded as cerebral ischemia-reperfusion (I/R) injury [6]. Due to the limited therapeutic interventions for cerebral I/R injury in clinic, it is necessary to research and develop new effective drugs to treat cerebral I/R injury.

Accumulating studies revealed that cerebral I/R injury involves a series of pathological events such as inflammation, oxidative stress, and metabolic dysfunction [6, 7]. Notably, there is growing evidence that glycolysis is closely related to the pathological process of stroke [8–10]. Under physiological conditions, neurons mainly use most of the glucose to produce ATP through the pentose-phosphate pathway (PPP), while the glycolysis state is low [11]. When cerebral I/R occurs, neuronal glucose metabolism converts from PPP to glycolysis, which is induced by the increased expression of the glycolytic promoting enzymes such as lactate dehydrogenase A (LDHA), glucose transporter1 (GLUT1), and hexokinase2 (HK2) [11, 12]. Enhancement of glycolysis also can lead to higher glucose consumption, more lactic acid accumulation, and less ATP production, which ultimately accelerates neuronal death [13]. It is reported that attenuating hyperglycolysis by inhibition of PFKFB3 activity resulted in the reduction of NADPH oxidation, redox stress, and apoptotic cell death in oxygen and glucose-deprived primary neurons [14]. Therefore, the inhibition of glycolysis may represent an effective option for the treatment of cerebral I/R injury.

Celastrol (3-hydroxy-24-nor-2-oxo-1(10), 3,5,7-friedelate-traen-29-oic acid), a pentacyclic-triterpene isolated from the herbal medicine *Tripterygium wilfordii* Hook, has been reported to have therapeutic potential for central nervous system diseases [15–18]. Several researches suggested that celastrol could protect against ischemic brain injury through its several pharmacological properties such as antioxidation, antiapoptosis, and anti-inflammatory [18–20]. Meanwhile, two studies suggested that celastrol could work as a leptin sensitizer to regulate energy homeostasis and improve glucose tolerance [21, 22], and another study also indicated that celastrol can inhibit glycolysis metabolic signalling in T helper 17 (Th17) cells [23]. These investigations suggested the metabolic regulating potential of celastrol in differential pathophysiological procedures. However, the protection of celastrol in cerebral I/R injury by regulating glucose metabolism has not yet been reported.

Hypoxia inducible factor-1*α* (HIF-1*α*) is an essential factor regulating cellular responses to hypoxia, and it participates in multiple pathological processes including metabolic disorders through binding to the hypoxia response elements (HREs) in the promoters and enhancers of numerous genes [24]. HIF-1*α* also works as a vital regulator following cerebral ischemic reperfusion injury, which is accompanied with decreased ATP, increased ROS, and mitochondrial-glycolytic shift in cellular energetics and metabolism [25]. Furthermore, PDK1 (pyruvate dehydrogenase kinase1), a direct target gene of HIF-1*α*, can inhibit the conversion of pyruvate to acetyl CoA, thereby decreasing glucose oxidation and increasing glycolytic metabolism [26, 27]. In addition, researchers have suggested that activation of HIF-1*α*/PDK1 axis can enhance glycolysis and ultimately aggravate cell injury [28]. And celastrol also can inhibit expression of HIF-1*α* in human hepatoma cell to produce anticancer effect [29]. Therefore, we hypothesized that the neuroprotection of celastrol in cerebral I/R injury may be partly through inhibiting HIF-1*α*/PDK1 to regulate glucose metabolism.

Herein, via a transient middle cerebral artery occlusion mice model, we revealed that celastrol provide neuroprotection in cerebral I/R injury through its metabolic regulating properties. After the I/R injury onset, celastrol could inhibit glycolysis through the HIF-1*α*/PDK1 axis. These findings proved our hypothesis and provide a new therapeutic potential of celastrol in ischemia/reperfusion injury.

## 2. Materials and Methods

### 2.1. Animals

Male C57BL/6 mice (18-22 g) were purchased from the Laboratory Animal Center of Chongqing Medical University. All mice were kept in a SPF-level laboratory under controlled conditions (12 h/12 h light/dark cycle, humidity 60 ± 10%, and temperature 24 ± 2°C) and allowed free access to food and water. The experiments were approved by the Animal Laboratory Administrative Center and the Institutional Ethics Committee of Chongqing Medical University (license number: SYXK (Chongqing) 2018-0003).

### 2.2. Establishment of Transient Middle Cerebral Artery Occlusion (tMCAO) Mice Model

The cerebral I/R injury animal model was established by the tMCAO surgery as our previous report [30, 31]. In short, animals were anesthetized with isoflurane (5% for induction and 1.5%-2.5% for maintenance). Then, a median incision was made in the neck; the right common carotid artery (CCA), internal carotid artery (ICA), and external carotid artery (ECA) were gently isolated. Subsequently, a 6-0 silicon-coated nylon suture (Yushun Biotech, Henan, China) was inserted from the ECA to the ICA until it occluded the origin of the right middle cerebral artery (MCA). After 60 min of MCAO, the suture was removed gently to allow reperfusion for 24 h. Mice in the sham group had the same surgery except occluding the MCA. Before, during, and after the MCAO surgery, Laser Doppler Flowmetry (Periflux System 5000, Perimed, Järfälla, Sweden) was applied to continuously measure the cerebral blood flow (CBF) of mice. A decrease in CBF ≥ 80% of baseline in MCA area after MCAO surgery and a recovery in CBF ≥ 70% of baseline within 10 min after removing the suture were considered to successfully induce cerebral I/R injury; otherwise, the mice will be excluded. Keep the body temperature of animals between 36.5°C and 37.5°C during the surgery and returned them to the cages until they were fully awake.

### 2.3. Groups and Drug Treatment

The mice were randomly divided into 7 groups: sham group: mice received equal volume of vehicle (0.9% saline solution containing 1% DMSO); I/R group: mice received equal volume of vehicle; I/R + Celastrol (3 mg/kg) group: mice received 3 mg/kg celastrol; I/R + Celastrol (4.5 mg/kg) group: mice received 4.5 mg/kg celastrol; I/R + Celastrol (6 mg/kg) group: mice received 6 mg/kg celastrol; I/R + Celastrol + DMOG (HIF-1*α* agonist, MedChemExpress, United States) group: mice received 4.5 mg/kg celastrol and 50 mg/kg DMOG; and I/R + DMOG group: mice received 50 mg/kg DMOG. Mice in the sham group were subjected to sham surgery, and mice in other groups underwent tMCAO surgery. Celastrol was dissolved in 0.9% saline solution containing 1% DMSO, and DMOG was dissolved in 0.9% saline solution. When the Laser Doppler Flowmetry detected the successful reperfusion of the CBF within 10 min after removing the suture, vehicle, celastrol, or DMOG was injected intraperitoneally immediately.

### 2.4. Evaluation of Neurological Score and Cerebral Infarct Volume

Cerebral I/R injury induced by tMCAO led to neurological dysfunction in experimental animals. The neurological deficit score can be used to assess the neurological dysfunction severity. After 1 h MCAO and 24 h reperfusion, the neurological deficit score was evaluated according to the modified neurological severity score (mNSS) by an investigator blinded to the groups (*n* = 4 − 5). After evaluation of neurological score, mice were sacrificed after anesthesia, and the brains were rapidly removed and frozen for 30 min at -20°C. After that, the brain tissue was slightly sliced into 5 coronal sections (3 mm thick) and incubated in 2% 2,3,5-triphenyltetrazolium chloride (TTC) solution at 37°C for 20 min, followed by fixation with 4% paraformaldehyde at 4°C overnight. Finally, the infarct tissue appeared white, and noninfarct tissue appeared red. The ImageJ software (National Institutes of Health, MD, USA) was used to measure the infarct size, and the cerebral infarct volume was calculated using the following formula: (contralateral hemisphere area − ipsilateral nonischemic hemisphere area)/contralateral hemisphere area × 100%.

### 2.5. Histopathological Examination

Hematoxylin-eosin (HE) staining was used to determine the histopathological damage in cerebral cortex. Briefly, 24 h after reperfusion, 3 mice in each group were deeply anesthetized with sodium pentobarbital (40 mg/kg) and perfused with PBS for 10 min, followed by perfusion with 4% paraformaldehyde for 10 min. After that, the brains were removed and stored in 4% paraformaldehyde at 4°C overnight. Then, the brain tissues were embedded in paraffin wax and coronal sliced into 5 *μ*m-thick sections. Finally, the coronal sections were stained with hematoxylin-eosin (HE) according to the manufacturer's protocol (Servicebio, Wuhan, China). The cortical cells were observed under a light microscopy at 200× and 400× magnifications.

### 2.6. Quantitative Real-Time PCR (qRT-PCR)

qRT-PCR was used to determine the mRNA level of HIF-1*α*, PDK1, GLUT1, HK2, and LDHA in cerebral cortex. TRIzol reagent (Vazyme, Nanjing, China) was used to extract total RNA from the cortical tissue following the manufacturer's instruction. The purity and concentration of RNA were determined by ultraviolet spectrophotometry. Reverse transcription of RNA was performed using the Reverse Transcriptase kit (Bimake, Houston, TX, USA). QRT-PCR was conducted to analyze the gene expression using the primer (Sangon Biotech, Shanghai, China) and SYBR Green II (Bimake, Houston, TX, USA). All data were normalized by *β*-actin. The primer sequences are shown in Table 1.

### 2.7. Western Blot Analysis

Western blot was used to detect the protein expression of HIF-1*α*, PDK1, GLUT1, LDHA, and HK2 in the cerebral cortex. The protein concentration was determined using a Bradford Protein Quantification Kit (Vazyme, Nanjing, China). An equal amount of protein was separated by 10% sodium dodecyl sulfate-polyacrylamide gel electrophoresis (SDS-PAGE) and transferred to the PVDF membranes (Millipore, USA). Afterwards, the membranes were blocked with 5% BSA at room temperature for 2 h and then incubated with specific primary antibodies against HIF-1*α* (dilution 1 : 1000, Abcam, United Kingdom), PDK1 (dilution 1 : 1000, Abcam, United Kingdom), GLUT1 (dilution 1 : 1000, Bioss, Beijing, China), LDHA (dilution 1 : 1000, GeneTex, United States), HK2 (dilution 1 : 1000, CST, United States), and *β*-actin (dilution 1 : 2000, ProteinTech, United States) overnight at 4°C. Subsequently, the membranes were incubated with horseradish peroxidase-conjugated secondary antibody (dilution 1 : 1000, ProteinTech, Wuhan, China) for 1 h at room temperature. Finally, the expression of target proteins was measured by enhanced chemiluminescence method (Bio-Rad, USA).

### 2.8. Glucose Content, ATP Level, and Lactate Production Assays

After 24 h reperfusion, the brains of the mice in each group were removed and rinsed with 0.9% saline solution; then, the cortex was separated and stored at -80°C. Glucose content (Solarbio, Beijing, China), ATP level (Beyotime Biotech, Shanghai, China), and lactate production (Nanjing Jiancheng Bioengineering Institute, Nanjing, China) in ipsilateral cortical tissue were detected by assay kits according to the manufacturer's instruction.

### 2.9. Statistical Analysis

The data were expressed as mean ± SEM and analyzed using GraphPad Prism 6.0 Software. Statistical significance was evaluated by one-way analysis of variance (ANOVA) followed by Tukey's multiple comparison test; *P* < 0.05 was considered statistically significant.

## 3. Results

### 3.1. Celastrol Improved Neurological Deficits, Reduced Infarct Volume, and Ameliorated Cortex Histopathological Damage in tMCAO Mice

**Figure 1:**
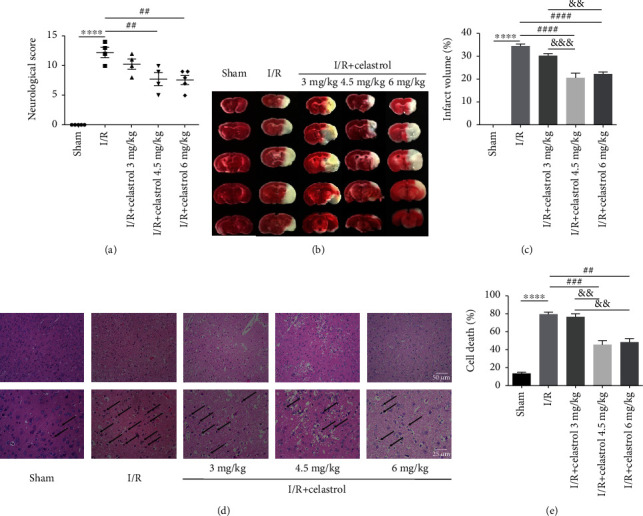
Effects of different doses of celastrol on cerebral I/R injury. (a) Neurological score in each group (*n* = 4 − 5). (b) Representative TTC staining of brain sections among different groups at 24 h after reperfusion. (c) Statistical analysis of cerebral infarct volume (*n* = 4 − 5). (d) HE staining of the ipsilateral cortex at 24 h after reperfusion. (e) Statistical analysis of dead cells in cortex (magnification: 400×) (*n* = 3). The data are presented as mean ± SEM. ^∗∗∗∗^*P* < 0.0001 compared with the sham group; ^##^*P* < 0.01, ^###^*P* < 0.001, and ^####^*P* < 0.0001 compared with the I/R group; ^&&^*P* < 0.01 and ^&&&^*P* < 0.001 compared with the I/R + 3 mg/kg Celastrol group.

To determine whether celastrol can attenuate cerebral I/R injury, cerebral infarct volume and neurological score were evaluated at 24 h after reperfusion. As shown in Figure 1(a), mice in the sham group showed no neurological deficits, while the neurological score of tMCAO mice was significantly increased, indicating that cerebral I/R caused severe neurological dysfunction. Compared with I/R group, 3 mg/kg celastrol had no obvious effect on improving neurological function, 4.5 and 6 mg/kg celastrol remarkably reduced neurological score, and there were no statistical differences between these two groups. Besides, the results of TTC staining showed there was no cerebral infarction observed in the sham group, while the cerebral infarct volume was obviously increased in mice subjected to tMCAO. Compared with the I/R group, 3, 4.5, and 6 mg/kg celastrol significantly reduced tMCAO-induced cerebral infarction. Among these three doses, 4.5 and 6 mg/kg celastrol had the similar effect and were better than 3 mg/kg celastrol in reducing cerebral infarct volume (Figures 1(b) and 1(c)). Therefore, 4.5 and 6 mg/kg celastrol had the optimal curative effect on the treatment of cerebral I/R injury. As shown by HE staining, the cortical cells in the sham group were closely arranged, and the cell structure was clear, while the damaged cells in cortex were disorderly arranged and appeared edema and rupture in the I/R group. Compared with the I/R group, the histopathological damage of cortical cells was reduced by 3, 4.5, and 6 mg/kg celastrol (Figure 1(d)). Moreover, 4.5 and 6 mg/kg celastrol could significantly reduce the I/R-induced cell death in cortex, and the protective effect of 4.5 and 6 mg/kg celastrol on ischemic tissues was equivalent and more obvious than 3 mg/kg celastrol (Figure 1(e)). These findings further clarified that celastrol was able to ameliorate cerebral I/R injury. To balance the toxicity and effect of celastrol [32], 4.5 mg/kg celastrol was selected for the subsequent research.

### 3.2. Celastrol Inhibited Cerebral I/R-Induced Glycolysis

**Figure 2:**
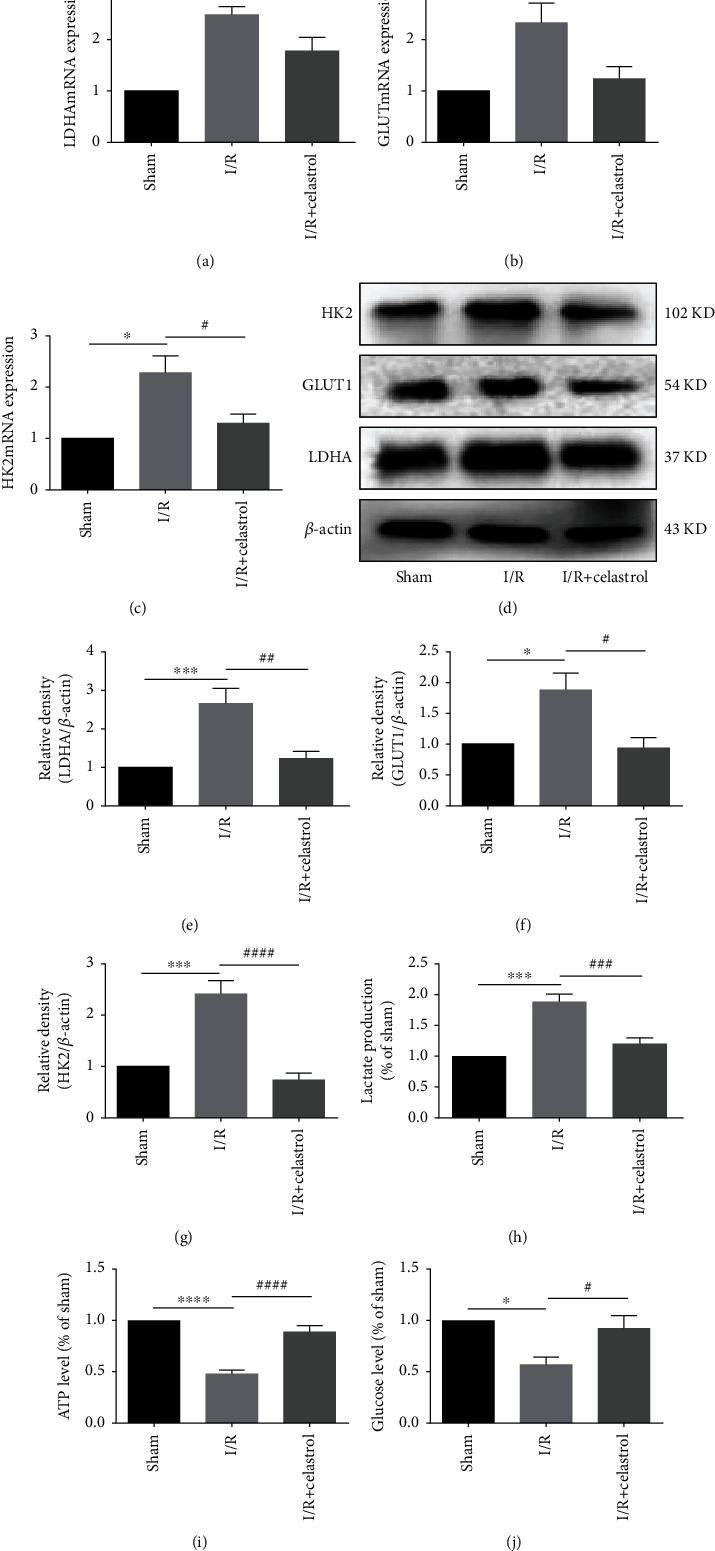
Celastrol decreased the level of glycolysis-related factors in ischemic cerebral cortex. QRT-PCR was used to detect the mRNA expression of LDHA (a), GLUT1 (b), and HK2 (c) (*n* = 4). Western blot was used to measure the protein expression of LDHA (d, e), GLUT1 (d, f), and HK2 (d, g) (*n* = 5). Lactate production (h), ATP level (i), and glucose content (j) were shown in the graphs, respectively (*n* = 5). The data are presented as mean ± SEM. ^∗^*P* < 0.05, ^∗∗^*P* < 0.01, ^∗∗∗^*P* < 0.001, and ^∗∗∗∗^*P* < 0.0001 compared with the sham group; ^#^*P* < 0.05, ^##^*P* < 0.01, ^###^*P* < 0.001, and ^####^*P* < 0.0001 compared with the I/R group.

In order to explore the influence of celastrol on glycolysis of ischemic brain tissue in tMCAO mice, we determined the mRNA and protein expression of glycolysis-related factors, such as LDHA, GlUT1, and HK2 in ischemic cortex. As demonstrated in Figures 2(a)–2(c), the mRNA expression of LDHA, HK2, and GlUT1 was significantly increased in the I/R group compared to the sham group. Compared with the I/R group, the above glycolysis-related factors showed notably decreased mRNA expression in the I/R + Celastrol group. Consistently, at the protein level, LDHA, GlUT1, and HK2 in cerebral cortex were expressed higher in the I/R group than those in the sham group, while celastrol remarkably downregulated the expression of these proteins in tMCAO mice (Figures 2(d)–2(g)). To further clarify the role of celastrol in regulating glycolysis, the lactate production, ATP level, and glucose content were evaluated. Compared with the sham group, the lactate production was significantly increased, and the ATP level as well as glucose content were significantly decreased in the I/R group, while celastrol treatment blocked these changes in the above indicators after cerebral I/R (Figures 2(h)–2(j)). These data suggested that celastrol can inhibit glycolysis in tMCAO mice.

### 3.3. Celastrol Downregulated the Expression of HIF-1*α* and PDK1 in tMCAO Mice

**Figure 3:**
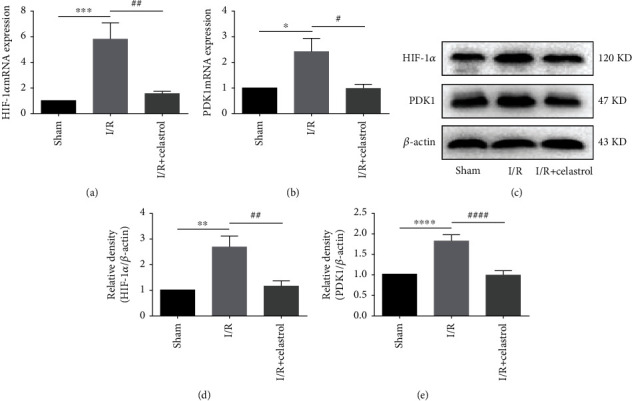
Celastrol suppressed the expressions of HIF-1*α* and PDK1 in the ischemic cerebral cortex. QRT-PCR was used to detect the mRNA expression of HIF-1*α* (a) and PDK1 (b) (*n* = 4). Western blot was used to measure the protein expression of HIF-1*α* (c, d) and PDK1 (c, e) (*n* = 5). The data are presented as mean ± SEM. ^∗^*P* < 0.05, ^∗∗^*P* < 0.01, ^∗∗∗^*P* < 0.001, and ^∗∗∗∗^*P* < 0.0001 compared with the sham group; ^#^*P* < 0.05, ^##^*P* < 0.01, and ^####^*P* < 0.0001 compared with the I/R group.

The HIF-1*α*/PDK1 axis plays an important role in regulating glucose metabolism. Therefore, the regulatory effect of celastrol on HIF-1*α*/PDK1 axis after cerebral I/R was investigated. As shown in Figures 3(a)–3(e), the mRNA and protein expression of HIF-1*α* and PDK1 was significantly upregulated in the I/R group when compared with the sham group. After treatment with celastrol, the expression of HIF-1*α* and PDK1 in ischemic cortex was remarkably downregulated at the mRNA and protein level, which preliminarily indicated that the HIF-1*α*/PDK1 axis may be associated with the effect of celastrol against cerebral I/R injury.

### 3.4. DMOG Blocked the Celastrol-Induced HIF-1*α* and PDK1 Downregulation in tMCAO Mice

**Figure 4:**
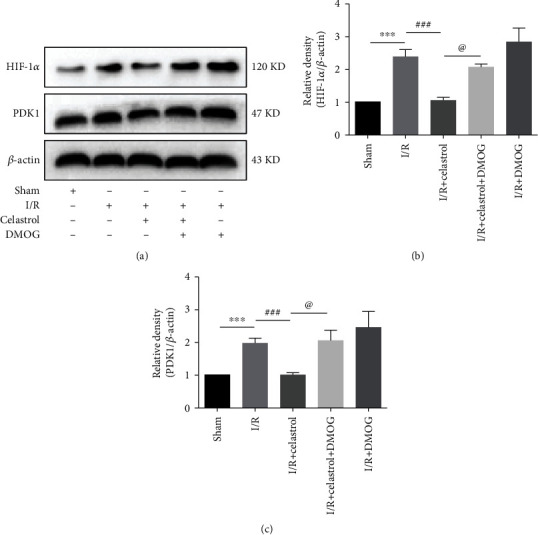
DMOG blocked celastrol-induced downregulation of HIF-1*α* and PDK1 in the ischemic cortex of tMCAO mice. Protein expressions of HIF-1*α* (a, b) and PDK1 (a, c) in each group are shown in the graphs (*n* = 5). The data are presented as mean ± SEM. ^∗∗∗^*P* < 0.001 compared with the sham group; ^###^*P* < 0.001 compared with the I/R group; ^@^*P* < 0.05 compared with the I/R + Celastrol group.

Figures 4(a)–4(c) show that the expression of HIF-1*α* and PDK1 in the I/R + DMOG group was higher than that in the I/R group, which suggested that DMOG effectively activated the HIF-1*α*/PDK1 axis. Celastrol was found to inhibit the upregulation of HIF-1*α* and PDK1 induced by cerebral ischemia in the I/R + Celastrol group as compared with the I/R group. Additionally, compared with the I/R + Celastrol group, the expression of HIF-1*α* and PDK1 was significantly upregulated in the I/R + Celastrol + DMOG group, revealing that the HIF-1*α*/PDK1 axis may be involved in the mechanism of celastrol in relieving cerebral I/R injury.

### 3.5. DMOG Reversed the Protective Effect of Celastrol on Cerebral I/R Injury

**Figure 5:**
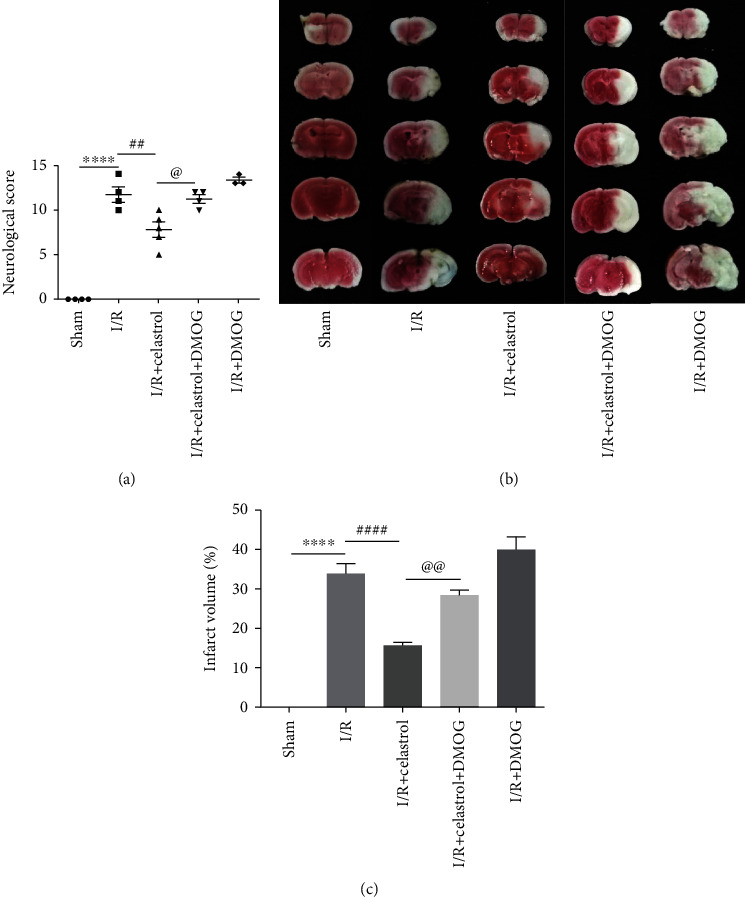
DMOG reversed the neuroprotective effect of celastrol on cerebral I/R injury. (a) Neurological score in different groups (*n* = 3 − 4). (b) Representative images of TTC staining. (c) Quantification of cerebral infarct volume in different groups (*n* = 3 − 4). The data are presented as mean ± SEM. ^∗∗∗∗^*P* < 0.0001 compared with the sham group; ^##^*P* < 0.01 and ^####^*P* < 0.0001 compared with the I/R group; ^@^*P* < 0.05 and ^@@^*P* < 0.001 compared with the I/R + Celastrol group.

DMOG, a specific agonist of HIF-1*α*, was employed to upregulate the HIF-1*α* expression to study the relationship between the neuroprotective effect of celastrol on cerebral I/R injury and its regulation of the HIF-1*α*/PDK1 axis. The results of neurological score indicated that the neurological score was increased in the I/R + DMOG group as compared with the I/R group, but not significant. Compared with the I/R group, celastrol obviously improved tMCAO-induced neurological deficits in the I/R + Celastrol group, but this effect was reversed by DMOG in the I/R + Celastrol + DMOG group (Figure 5(a)). In addition, Figures 5(b) and 5(c) show that a larger cerebral infarction was observed in the I/R + DMOG group compared to the I/R group, suggesting that the upregulation of HIF-1*α* in ischemic brain tissue may aggravate cerebral I/R injury. Furthermore, treatment with celastrol significantly reduced cerebral infarct volume after ischemic brain injury, while the infarct volume showed significant increase in the I/R + Celastrol + DMOG group when compared with the I/R + Celastrol group. The above data demonstrated that DMOG-induced upregulation of HIF-1*α* reversed the protective effect of celastrol on cerebral I/R injury.

### 3.6. DMOG Abolished the Celastrol-Induced Inhibition of Glycolysis in tMCAO Mice

**Figure 6:**
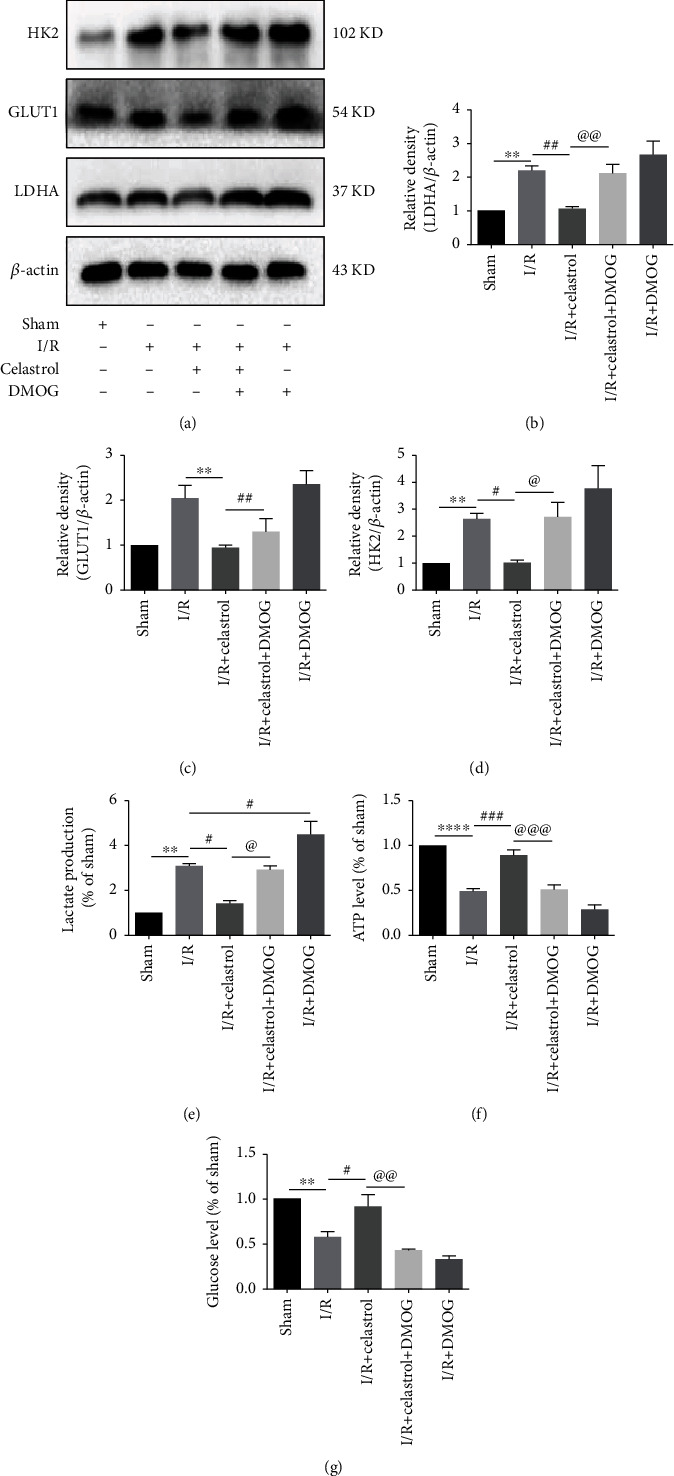
DMOG blocked celastrol-induced suppression of glycolysis. The protein expression of glycolysis-related factors including LDHA (a, b), GLUT1 (a, c), and HK2 (a, d) in ipsilateral cortex were detected (*n* = 5). Qualification analysis of lactate production (e), ATP level (f), and glucose content (g) in different groups (*n* = 5). The data are presented as mean ± SEM. ^∗∗^*P* < 0.01 and ^∗∗∗∗^*P* < 0.0001 compared with the sham group; ^#^*P* < 0.05, ^##^*P* < 0.01, and ^###^*P* < 0.001 compared with the I/R group; ^@^*P* < 0.05, ^@@^*P* < 0.01, and ^@@@^*P* < 0.001 compared with I/R + Celastrol group.

To further explore the mechanism underlying the neuroprotection of celastrol on cerebral I/R injury, we examined the expression of glycolysis-related factors in ischemic cortex after DMOG treatment. The results demonstrated that the expression of LDHA, GLUT1, and HK2 was higher in the I/R + DMOG group than those in the I/R group, but not statistically significant. Compared with the I/R + Celastrol group, the LDHA and HK2 expression was notably upregulated, and the GLUT1 expression showed tendency of increase in the I/R + Celastrol + DMOG group (Figures 6(a)–6(d)). Furthermore, as illustrated in Figures 6(e)–6(g), the lactate production was significantly elevated, and the ATP level and glucose content showed tendency of decreased in the I/R + DMOG group compared to the I/R group, which suggested the activation of the HIF-1*α*/PDK1 axis can enhance glycolysis in cerebral I/R injury. Additionally, I/R-induced glycolysis can be inhibited by celastrol treatment, as the I/R + Celastrol group showed decreased lactate production and increased ATP level and glucose content as compared with the I/R group. Simultaneously, compared with the I/R + Celastrol group, DMOG partially reversed the inhibitory effect of celastrol on glycolysis in the I/R + Celastrol + DMOG group. Taken together, our results confirmed that celastrol protected against cerebral I/R injury through suppressing glycolysis via targeting the HIF-1*α*/PDK1 axis in tMCAO mice.

## 4. Discussion and Conclusions

In current study, we aimed to investigate the glucose metabolism-regulating effect of celastrol on cerebral ischemic reperfusion injury and the underlying mechanism. Wild-type mice were subjected to MCAO followed by 24-hour reperfusion. Celastrol could significantly reduce the infarct volume and neurologic deficits after one intraperitoneal injection at the onset of reperfusion. We found that celastrol decreased the mRNA and protein expression of three vital enzymes of glycolysis, LDHA, HK2, and Glut1. As a result, celastrol decreased the lactate production and increased the ATP and glucose level. Furthermore, our results found that celastrol treatment inhibited the mRNA and protein expression of HIF-1*α* and PDK1, which may indicate that celastrol could regulate glycolysis via HIF-1*α*/PDK1. We adopted DMOG, a HIF-1*α* agonist, to further confirm our hypothesis. And we proved that DMOG could reverse the neuroprotection of celastrol and its effect on regulating glycolysis through inhibiting the expression of HIF-1*α*/PDK1 and glycolysis enzymes including LDHA, HK2, and Glut1. In summary, our results suggested that celastrol could regulate glucose metabolism against cerebral ischemic reperfusion injury through inhibiting HIF-1*α*/PDK1.

Celastrol, the most abundant bioactive constituents of *Tripterygium wilfordii* Hook F (TWHF), plays a protective role in many neurological disease models [17, 33]. Several studies have reported that celastrol can protect against stroke by its anti-inflammatory and antiapoptotic effects, which were relative to promoting IL-33/ST2-mediated M2 polarization of microglia and downregulating p-JNK, p-c-Jun, and NF-*κ*B [18, 20]. In accordance with the previous studies, we also confirmed the neuroprotection of celastrol in cerebral ischemic reperfusion injury. But are there other mechanisms related with neuroprotection of celastrol except anti-inflammation, antioxidation, and antiapoptosis? Since 2015, the leptin-sensitization effect of celastrol has been proposed in treating obesity [22]; further study found that celastrol exhibits a strong antidiabetic property in mice through sensitizing endogenous leptin [34]. Subsequently, the metabolic regulating effect of celastrol, especially its role in glucose metabolism, attracted increasingly attention. Further investigations also provide evidences that celastrol could regulate glucose metabolism through PGC-1*α*/GLUT4, HO-1, and Gas6 (growth arrest-specific 6) pathways, respectively [35–37]. Therefore, we extrapolated that celastrol may act as a glucose metabolic regulator to produce its neuroprotective effect in cerebral ischemic reperfusion injury.

Glucose acts as a double-edge sword in stroke and ischemic-reperfusion injury. The dramatic variation of glucose and oxygen during cerebral ischemia results in ATP depletion and then leads to the neuronal cell death. Although glycolysis could produce ATP in the absence of oxygen via glucose, current experimental and clinical evidences suggest a negative effect in the ischemic reperfusion injury [38]. Thus, regulating the glucose metabolism may be a promising strategy to alleviate the I/R-induced injury. In ischemic/reperfused heart model, suppression of mTORC1-dependent glycolysis by salvianolic acid B can effectively reduce inflammation and improve cardiac dysfunction [39]. Cai et al. reported that enhanced glycolysis aggravate cerebral I/R injury after 24-hour reperfusion via the YY1/lncRNA GAS5 complex axis [11]. Another study also suggested that there is a significant increase in glycolysis in ischemic brain tissue at 24 h of reperfusion, and dichloroacetic acid could produce neuroprotection by regulating poststroke glycolysis via inhibiting PDK2 and activating PDH [40]. Our present results showed that cerebral I/R-induced glycolysis was significantly inhibited by celastrol at 24 h after reperfusion, which was evidenced by lower glucose consumption, less lactate accumulation, more ATP production, and downregulations of glycolysis-related key enzymes including LDHA, HK2, and Glut1. These results revealed that the neuroprotective effect of celastrol on cerebral I/R injury was closely associated with inhibition of glycolysis in the first 24 hours, but the effect of celastrol on glycolysis at different stages of cerebral ischemia and the glycolysis-regulating effect to the outcome of I/R injury still need further research to unveil.

HIF-1 is a heterodimer consisting of a constitutive HIF-1*β* subunit and an inducible HIF-1*α* subunit, in which an HIF-1*α* subunit primarily determines HIF-1 activation and regulates various genes to participate in apoptosis, autophagy, angiogenesis, and glucose metabolic dysfunction [41, 42]. Inhibition of HIF-1*α* was reported to suppress inflammasome, reduce apoptotic cell death, improve blood brain barrier damage, etc. in stroke-induced brain injury, through LDHA (lactate dehydrogenase), matrix metalloproteinase-2 (MMP-2), vascular endothelial growth factor (VEGF), and so on [43–45]. Significantly, a latest study found that inhibition of HIF-1*α*-mediated hyperglycolysis by chlorpromazine and promethazine (C+P) can effectively protect against ischemic stroke injury [46]. Moreover, inhibition of HIF-1*α* by celastrol has been confirmed in rheumatoid arthritis (RA) joints and liver cancer [29, 47], but there was no relevant research in stroke. Thus, we proposed that celastrol may target on HIF-1*α*-meditated glycolysis to protect against cerebral I/R injury. Consistent with our hypothesis, the results showed that celastrol treatment significantly decreased the expression of HIF-1*α* and upregulation of HIF-1*α* by DMOG that can block the inhibition of celastrol on I/R-induced hyperglycolysis, which suggested that celastrol protected I/R-induced brain injury by inhibiting HIF-1*α*-mediated glycolysis.

As a direct target gene of HIF-1*α*, PDK1 can decrease glucose oxidation through inactivating pyruvate dehydrogenase (PDH), which converts pyruvate to acetyl CoA [26]. Previous studies reported that activation of HIF-1*α* through specific pathways could increase PDK1 and trigger glycolytic metabolism, particularly during heart surgery or partial ischemia [48, 49]. For ischemic stroke, inhibition of stroke-induced activation of the HIF-1*α*/PDK1 axis can improve glucose metabolism which mitigates ischemic brain injury [50, 51]. Our study also showed that the expression of HIF-1*α* and PDK1 significantly increased in the I/R group, while celastrol treatment remarkably reduced them, which indicates that the HIF-1*α*/PDK1 axis may participate in the protective effect of celastrol in cerebral I/R injury. Administration of DMOG abolished the celastrol-induced reduction of HIF-1*α* and PDK1 expressions, indicating PDK1 is the downstream of HIF-1*α*, and further confirmed that celastrol could inhibit the HIF-1*α*/PDK1 axis in ischemic brain tissue. Altogether, the current study showed that the HIF-1*α*/PDK1 axis may play a critical role in the celastrol-induced neuroprotection by regulating glycolysis.

Although our study has demonstrated that celastrol can regulate glycolysis to exert neuroprotective effect via inhibition of the HIF-1*α*/PDK1 pathway in cerebral I/R injury, there still exist some problems. Firstly, this study was based on an in vivo experiment, and the specific cell type of celastrol acting on and the exact mechanism in the specific cell type still need further study to investigate. Secondly, the subsequent effect, like variation of inflammation and oxidative of HIF-1*α*/PDK1-regulated glycolysis, and the underlying mechanism also need to explore in the further research.

## 5. Conclusion

In summary, the current study proved that celastrol could ameliorate neurological deficits, cerebral infarction, and histopathological damage following cerebral I/R injury in mice. The mechanism of this neuroprotective effect may be related to the inhibition of glycolysis via targeting the HIF-1*α*/PDK1 axis. Our results provided experimental evidence that celastrol may be a potential candidate for the treatment of cerebral I/R injury.

## Figures and Tables

**Table 1:** Primer sequences of mRNA for RT-PCR analysis.

Gene	Forward sequence (5′ to 3′)	Reverse sequence (5′ to 3′)	Product size/bp
*Hif-1α*	ACATCAAGTCAGCAACGTGGAAGG	GCAAGCATCCTGTACTGTCCTGTG	379
*Pdk1*	ACTGCGACGTGGTGGAGGTC	ATGGTCGCTCTCATGGCATTCTTG	202
*Glut1*	CAGTTCGGCTATAACACTGGTG	GCCCCCGACAGAGAAGATG	156
*Ldha1*	TGTCTCCAGCAAAGACTACTGT	GACTGTACTTGACAATGTTGGGA	155
*Hk2*	TGATCGCCTGCTTATTCACGG	AACCGCCTAGAAATCTCCAGA	112
*Actb*	TGATCGCCTGCTTATTCACGG	AACCGCCTAGAAATCTCCAGA	205

## Data Availability

The data used to support the findings of this study are available from the corresponding authors upon request.

## References

[1] Avan A., Digaleh H., Di Napoli M. (2019). Socioeconomic status and stroke incidence, prevalence, mortality, and worldwide burden: an ecological analysis from the Global Burden of Disease Study 2017. *BMC Medicine*.

[2] Feigin V. L., Abajobir A. A., Abate K. H. (2017). Global, regional, and national burden of neurological disorders during 1990-2015: a systematic analysis for the Global Burden of Disease Study 2015. *The Lancet Neurology*.

[3] Benjamin E. J., Muntner P., Alonso A. (2019). Heart Disease and Stroke Statistics-2019 update: a report from the American Heart Association. *Circulation*.

[4] Anderson C. S., Robinson T., Lindley R. I. (2016). Low-dose versus standard-dose intravenous alteplase in acute ischemic stroke. *The New England Journal of Medicine*.

[5] Emberson J., Lees K. R., Lyden P. (2014). Effect of treatment delay, age, and stroke severity on the effects of intravenous thrombolysis with alteplase for acute ischaemic stroke: a meta- analysis of individual patient data from randomised trials. *The Lancet*.

[6] Wang S., Fu Y. Y., Han X. Y. (2021). Hyperbaric oxygen preconditioning protects against cerebral ischemia/reperfusion injury by inhibiting mitochondrial apoptosis and energy metabolism disturbance. *Neurochemical Research*.

[7] Lin L., Wang X., Yu Z. (2016). Ischemia-reperfusion injury in the brain: mechanisms and potential therapeutic strategies. *Biochem Pharmacol (Los Angel)*.

[8] Kadri S., el Ayed M., Kadri A., Limam F., Aouani E., Mokni M. (2021). Protective effect of grape seed extract and orlistat co-treatment against stroke: effect on oxidative stress and energy failure. *Biomedicine & Pharmacotherapy*.

[9] Wang X., Zhou Y., Tang D. (2019). ACC1 (acetyl coenzyme a carboxylase 1) is a potential immune modulatory target of cerebral ischemic stroke. *Stroke*.

[10] Vojinovic D., Kalaoja M., Trompet S. (2020). Association of circulating metabolites in plasma or serum and risk of Stroke. *Neurology*.

[11] Zhang X. C., Gu A. P., Zheng C. Y. (2019). YY1/LncRNA GAS5 complex aggravates cerebral ischemia/reperfusion injury through enhancing neuronal glycolysis. *Neuropharmacology*.

[12] Li T., Xian H. C., Dai L., Tang Y. L., Liang X. H. (2021). Tip of the iceberg: roles of circRNAs in cancer glycolysis. *Oncotargets and Therapy*.

[13] Cai L., Stevenson J., Peng C. (2016). Adjuvant therapies using normobaric oxygen with hypothermia or ethanol for reducing hyperglycolysis in thromboembolic cerebral ischemia. *Neuroscience*.

[14] Burmistrova O., Olias-Arjona A., Lapresa R. (2019). Targeting PFKFB3 alleviates cerebral ischemia-reperfusion injury in mice. *Scientific Reports*.

[15] Salminen A., Lehtonen M., Paimela T., Kaarniranta K. (2010). Celastrol: molecular targets of thunder god vine. *Biochemical and Biophysical Research Communications*.

[16] Zhao Y., Zhao H., Lobo N., Guo X., Gentleman S. M., Ma D. (2014). Celastrol enhances cell viability and inhibits Amyloid-*β* production induced by lipopolysaccharide in vitro. *Journal of Alzheimer’s Disease*.

[17] Choi B. S., Kim H., Lee H. J. (2014). Celastrol from ‘Thunder God Vine’ protects SH-SY5Y cells through the preservation of mitochondrial function and inhibition of p38 MAPK in a rotenone model of Parkinson's disease. *Neurochemical Research*.

[18] Jiang M., Liu X., Zhang D. (2018). Celastrol treatment protects against acute ischemic stroke-induced brain injury by promoting an IL-33/ST2 axis-mediated microglia/macrophage M2 polarization. *Journal of Neuroinflammation*.

[19] Zhang B., Zhong Q., Chen X. (2020). Neuroprotective effects of celastrol on transient global cerebral ischemia rats via regulating HMGB1/NF-*κ*B signaling pathway. *Frontiers in Neuroscience*.

[20] Li Y., He D., Zhang X. (2012). Protective effect of celastrol in rat cerebral ischemia model: Down-regulating p-JNK, p-c-Jun and NF-*κ*B. *Brain Research*.

[21] Ma X., Xu L., Alberobello A. T. (2015). Celastrol Protects against Obesity and Metabolic Dysfunction through Activation of a HSF1-PGC1*α* Transcriptional Axis. *Cell Metabolism*.

[22] Liu J., Lee J., Salazar Hernandez M. A., Mazitschek R., Ozcan U. (2015). Treatment of obesity with celastrol. *Cell*.

[23] Zhang J., Shan J., Chen X., Li S., Long D., Li Y. (2018). Celastrol mediates Th17 and Treg cell generation via metabolic signaling. *Biochemical and Biophysical Research Communications*.

[24] Hou Y., Wang J., Feng J. (2019). The neuroprotective effects of curcumin are associated with the regulation of the reciprocal function between autophagy and HIF-1&alpha; in cerebral ischemia-reperfusion injury. *Drug Design, Development and Therapy*.

[25] Ham P., Raju R. (2017). Mitochondrial function in hypoxic ischemic injury and influence of aging. *Progress in neurobiology*.

[26] Kim J. W., Tchernyshyov I., Semenza G. L., Dang C. V. (2006). HIF-1-mediated expression of pyruvate dehydrogenase kinase: a metabolic switch required for cellular adaptation to hypoxia. *Cell Metabolism*.

[27] Hong D. K., Kho A. R., Choi B. Y. (2018). Combined treatment with dichloroacetic acid and pyruvate reduces hippocampal neuronal death after transient cerebral ischemia. *Frontiers in Neurology*.

[28] Zhao H., Jiang H., Li Z. (2017). 2-Methoxyestradiol enhances radiosensitivity in radioresistant melanoma MDA-MB-435R cells by regulating glycolysis via HIF-1*α*/PDK1 axis. *International Journal of Oncology*.

[29] Ma J., Han L. Z., Liang H. (2014). Celastrol inhibits the HIF-1*α* pathway by inhibition of mTOR/p70S6K/eIF4E and ERK1/2 phosphorylation in human hepatoma cells. *Oncology Reports*.

[30] Cao J., Dong L., Luo J. (2021). Supplemental N-3 polyunsaturated fatty acids limit A1-specific astrocyte polarization via attenuating mitochondrial dysfunction in ischemic stroke in mice. *Oxidative Medicine and Cellular Longevity*.

[31] Luo C., Ouyang M. W., Fang Y. Y. (2017). Dexmedetomidine protects mouse brain from ischemia-reperfusion injury via inhibiting neuronal autophagy through up-regulating HIF-1*α*. *Frontiers in Cellular Neuroscience*.

[32] Hou W., Liu B., Xu H. (2020). Celastrol: progresses in structure-modifications, structure-activity relationships, pharmacology and toxicology. *European Journal of Medicinal Chemistry*.

[33] Veerappan K., Natarajan S., Ethiraj P., Vetrivel U., Samuel S. (2017). Inhibition of IKK*β* by celastrol and its analogues - anin silicoandin vitroapproach. *Pharmaceutical Biology*.

[34] Lee J., Liu J., Feng X. (2016). Withaferin A is a leptin sensitizer with strong antidiabetic properties in mice. *Nature medicine*.

[35] Fang P., He B., Yu M. (2019). Treatment with celastrol protects against obesity through suppression of galanin-induced fat intake and activation of PGC-1*α*/GLUT4 axis-mediated glucose consumption. *Biochimica et Biophysica Acta (BBA)-Molecular Basis of Disease*.

[36] Zhan X., Yan C., Chen Y. (2018). Celastrol antagonizes high glucose-evoked podocyte injury, inflammation and insulin resistance by restoring the HO-1-mediated autophagy pathway. *Molecular immunology*.

[37] Ma L., Cao Y., Zhang L. (2020). Celastrol mitigates high glucose-induced inflammation and apoptosis in rat H9c2 cardiomyocytes via miR-345-5p/growth arrest-specific 6. *The journal of gene medicine*.

[38] Robbins N., Swanson R. J. S. (2014). Opposing effects of glucose on stroke and reperfusion injury: acidosis, oxidative stress and energy metabolism. *Stroke*.

[39] Zhao M., Li F., Jian Y. (2020). Salvianolic acid B regulates macrophage polarization in ischemic/reperfused hearts by inhibiting mTORC1-induced glycolysis. *European Journal of Pharmacology*.

[40] Zhao X., Li S., Mo Y. (2021). DCA Protects against Oxidation Injury Attributed to Cerebral Ischemia- Reperfusion by Regulating Glycolysis through PDK2-PDH-Nrf2 Axis. *Oxidative Medicine and Cellular Longevity*.

[41] Kloska A., Malinowska M., Gabig-Cimińska M., Jakóbkiewicz-Banecka J. (2020). Lipids and lipid mediators associated with the risk and pathology of ischemic stroke. *International Journal of Molecular Sciences*.

[42] Reimann S., Fink L., Wilhelm J. (2015). Increased S100A4 expression in the vasculature of human COPD lungs and murine model of smoke-induced emphysema. *Respiratory research*.

[43] Chen S. F., Pan M. X., Tang J. C. (2020). Arginine is neuroprotective through suppressing HIF-1*α*/LDHA-mediated inflammatory response after cerebral ischemia/reperfusion injury. *Molecular Brain*.

[44] Shen Y., Gu J., Liu Z. (2018). Inhibition of HIF-1*α* reduced blood brain barrier damage by regulating MMP-2 and VEGF during acute cerebral ischemia. *Frontiers in Cellular Neuroscience*.

[45] Wang H., Xu X., Yin Y. (2020). Catalpol protects vascular structure and promotes angiogenesis in cerebral ischemic rats by targeting HIF-1*α*/VEGF. *Phytomedicine*.

[46] Guo S., Cosky E., Li F. (2021). An inhibitory and beneficial effect of chlorpromazine and promethazine (C+ P) on hyperglycolysis through HIF-1*α* regulation in ischemic stroke. *Brain Research*.

[47] Li G. Q., Liu D., Zhang Y. (2013). Anti-invasive effects of celastrol in hypoxia-induced fibroblast-like synoviocyte through suppressing of HIF-1*α*/CXCR4 signaling pathway. *International Immunopharmacology*.

[48] Roche T. E., Hiromasa Y. (2007). Pyruvate dehydrogenase kinase regulatory mechanisms and inhibition in treating diabetes, heart ischemia, and cancer. *Cellular and Molecular Life Sciences*.

[49] Semba H., Takeda N., Isagawa T. (2016). HIF-1*α*-PDK1 axis-induced active glycolysis plays an essential role in macrophage migratory capacity. *Nature Communications*.

[50] Zeng M., Zhou H., He Y. (2021). Danhong injection alleviates cerebral ischemia/reperfusion injury by improving intracellular energy metabolism coupling in the ischemic penumbra. *Biomedicine & Pharmacotherapy*.

[51] Zeng M., Shao C., Zhou H. (2021). Protocatechudehyde improves mitochondrial energy metabolism through the HIF1*α*/PDK1 signaling pathway to mitigate ischemic stroke-elicited internal capsule injury. *Journal of Ethnopharmacology*.

